# Transesophageal Echocardiography as a Monitoring Tool during Transvenous Lead Extraction—Does It Improve Procedure Effectiveness?

**DOI:** 10.3390/jcm9051382

**Published:** 2020-05-08

**Authors:** Dorota Nowosielecka, Wojciech Jacheć, Anna Polewczyk, Łukasz Tułecki, Konrad Tomków, Paweł Stefańczyk, Andrzej Tomaszewski, Wojciech Brzozowski, Dorota Szcześniak-Stańczyk, Andrzej Kleinrok, Andrzej Kutarski

**Affiliations:** 1 Department of Cardiology, The Pope John Paul II Province Hospital, 22-400 Zamosc, Poland; dornowos@wp.pl (D.N.); paolost@interia.pl (P.S.); a.kleinrok@wp.pl (A.K.); 2 2nd Department of Cardiology, Silesian Medical University, 41-800 Zabrze, Poland; wjachec@interia.pl; 3 Department of Physiology, Patophysiology and Clinical Immunology, Collegium Medicum of Jan Kochanowski University in Kielce, 25-317 Kielce, Poland; 4 Department of Cardiac Surgery, Swietokrzyskie Cardiology Center, 25-736 Kielce, Poland; 5 Department of Cardiac Surgery, The Pope John Paul II Province Hospital, 22-400 Zamosc, Poland; luke27@poczta.onet.pl (Ł.T.); konradtomkow@wp.pl (K.T.); 6 Department of Cardiology, Medical University, 20-400 Lublin, Poland; benecho2008@gmail.com (A.T.); brzozo@wp.pl (W.B.); dorotasstanczyk@gmail.com (D.S.-S.); a_kutarski@yahoo.com (A.K.); 7 University of Information Technology and Management, 35-959 Rzeszow, Poland

**Keywords:** transesophageal echocardiography, continuous intraprocedural monitoring, transvenous lead extraction

## Abstract

Background: Transesophageal echocardiography (TEE) is a valuable tool for monitoring the patient during transvenous lead extraction (TLE), but the direct impact of TEE on the effectiveness and safety of TLE has not yet been documented. Methods: The effectiveness of TLE and short-term survival were compared between two groups of patients: 2106 patients in whom TEE was performed before and after TLE and 1079 individuals in whom continuous TEE monitoring was used. The procedure-related risk of major complications was assessed using a predictive SAFeTY TLE score. Results: The patients monitored by TEE were characterized by older age, more comorbidities and higher SAFeTY TLE scores (6.143 ± 4.395 vs. 5.593 ± 4.127; *p* = 0.004). Complete procedural success was significantly higher in the TEE-guided group (97.683% vs. 95.442%, *p* < 0.01). The rate of serious complications in the TEE-guided group was lower than the predictive SAFeTY TLE score—a reduction of 28.75% (*p* < 0.05). Periprocedural mortality in the TEE-guided and non-TEE-guided groups was zero vs. six deaths (*p* = 0.186). Short-term survival was comparable between the groups. Conclusions: Transesophageal echocardiography as a monitoring tool during transvenous lead extraction provides valuable results—higher rates of complete procedural success and a reduced risk of the most severe complications, thus preventing periprocedural deaths.

## 1. Introduction

Transvenous lead extraction (TLE), as a method for managing complications related to cardiac implantable electronic devices (CIED), has been evolving since the 1990s and is now widely used in hospitals all over the world. The European Heart Rhythm Association (EHRA) report indicates that more than 9000 TLEs are performed annually at over 350 centers [1,2,3].

Lead extraction techniques involve manual traction or laser energy to dissect and remove lead from the cardiovascular system. Given the potential for serious complications related to cardiac tear or venous injury, it is crucial to constantly improve the safety of the procedure being performed. TLE belongs to relatively safe invasive procedures - the percentage of major complications range from 0.9 to 4.0%, unfortunately, procedural deaths (up to 1.86%) are still reported [4,5,6,7,8,9,10,11,12,13,14].

Both European and American guidelines published in 2017 provided more refined recommendations regarding the safety of TLE procedures. It is therefore recommended to perform TLE in an operating room or a hybrid operating room, preferably under general anesthesia, with direct systemic blood pressure monitoring via an indwelling arterial catheter, with a cardiothoracic surgeon available and with the continuous monitoring of the patient using transesophageal echocardiography (TEE) or intracardiac echocardiography (ICE) [13,14]. This study therefore sets out to assess the impact of continuous TEE monitoring on the effectiveness and safety of TLE. In order to estimate TLE-related risk, we use a previously devised SAFeTY TLE score to determine the probability of developing major complications during the procedure [15].

## 2. Methods

### 2.1. Study Population

This post-hoc analysis used clinical data of 3185 patients who underwent transvenous lead extraction between March 2006 and January 2020. Between the years 2006 and 2015, there were 2106 lead extractions with concomitant transthoracic echocardiography (TTE) and transesophageal echocardiography before and after the procedure, without intraprocedural monitoring. Between June 2015 and January 2020 there were 1079 lead extractions entirely under TEE guidance. All the procedures were performed at two different centers by the same experienced operator in both cases.

### 2.2. Lead Extraction Procedure

Lead extraction procedures were performed using mechanical systems such as polypropylene Byrd dilator sheaths (Cook^®^ Medical, Leechburg, PA, USA), mainly via the subclavian approach on the side of the implanted device. If technical difficulties arose, a different vascular access and/or additional tools such as Evolution (Cook^®^ Medical, Bloomington, IN, USA), TightRail (Spectranetix Colorado Springs, CO, USA), lassos and basket catheters were utilized. Laser cutting sheaths were not used. In the non-TEE-guided group, extraction procedures were performed by an experienced TLE operator and qualified nurses in an electrophysiology lab, in patients under general anesthesia and sedation. In the TEE-guided group lead extractions were performed by a team consisting of the same experienced TLE operator, with a second operator who had experience with pacing therapy, acardiac surgeon, anesthesiologist and echocardiographist. Procedures were performed in an operating room or in a hybrid operating room, in patients under general anesthesia, with continuous invasive blood pressure monitoring. Indications for TLE and type of periprocedural complications were defined according to the *2017* HRS Expert Consensus Statement on Cardiovascular Implantable Electronic Device Lead Management and Extraction *a*s well as the European Lead Extraction ConTRolled (ELECTRa) study: a European Heart Rhythm Association (EHRA) Registry of Transvenous Lead Extraction Outcomes [13,14].

### 2.3. Transesophageal Echocardiographic Monitoring

Transesophageal echocardiographic monitoring was performed using the GE Vivid 3, Vivid 4, and Vivid S 70 systems, as well as the Philips iE33 ultrasound machine equipped with transesophageal probes for 3D and 4D imaging. All images were stored in a digital memory for extended analysis after the procedure. Leads were assessed in standard esophageal and transgastric views. Standard views included mid-esophageal views (0°, 30°–35°, 60°–75°, 80°–100°, 100°–150°) visualizing the right atrium, tricuspid valve, right ventricular outflow tract (RVOT) and right ventricular inflow tract (RVIT); low esophageal views (0°, 45°–55°, 80°–90°) visualizing the coronary sinus, tricuspid valve; and transgastric right ventricular views (30°–40°, 90°, 100°–110°, 110°–130°) visualizing three leaflets of the tricuspid valve, right ventricular long axis, RVOT, and right ventricular cross-sections. Sometimes, if necessary, nonconventional imaging planes were used for better visualization of the structures.

TEE monitoring was conducted in three phases: preprocedural, intraprocedural and postprocedural. The study started after patient intubation with the insertion of an echocardiographic probe down the esophagus. All study phases were performed during the same extraction procedure. In the preprocedural phase, we evaluated the lead position, degree of fibrous encapsulation, lead-to-lead adhesions, unaccounted-for structures in the leads, as well as the function of the tricuspid valve and the pericardium. The intraprocedural phase was performed during the extraction procedure, taking into account direct pulling on the cardiac walls, obliterating the right ventricular lumina and their effect on the hemodynamic status of the patient. Furthermore, we observed pulling on other leads, detachment and dislodgement of fibrous tissue or vegetation fragments, and the separation of pericardial layers. The postprocedural phase included an evaluation of tricuspid valve function, remnants of masses removed during TLE (vegetations, fibrous tissue), and the abnormal accumulation of fluid in the pericardial sac.

### 2.4. Estimating the Number of Major Complications Using the SAFeTY TLE Score 

The SAFeTY TLE score assesses the risk of the occurrence of major complications related to TLE and takes into account the following parameters: sum of dwell times of extracted leads (threshold value ≥16.5 years), hemoglobin level in the blood (threshold level <11.5 g/dL), female gender, the number of previous CIED-related procedures and age below 30 years at first CIED implantation. The numbers of expected major complications in the two groups were determined using the SAFeTY TLE score calculator, an online tool available at http://alamay2.linuxpl.info/kalkulator/ [15].

### 2.5. Statistical Analysis

The Shapiro–Wilk test showed that most continuous variables were normally distributed. For uniformity, all continuous variables are presented as the mean ± standard deviation. The categorical variables are presented as a number and percentage. The significance of differences between groups was determined using the nonparametric chi^2^ test with Yates correction or the unpaired “U” Mann–Whitney test, as appropriate.

The Kaplan–Meier method was used to calculate the probability of mortality-free survival in patients divided into two groups according to the presence or absence of TEE monitoring during TLE. The log-rank test, including complete and censored data, was used to test for differences between the survival curves. A two-tailed *p* value < 0.05 was considered statistically significant. Statistical analysis was performed with Statistica version 13.3 (TIBCO Software Inc., AP Palo Alto, CA, USA).

## 3. Results

### 3.1. Clinical Characteristics of the Study Population

TEE-guided patients were older and more frequently had comorbidities (Charlson comorbidity index 4.972 ± 3.756 vs. 4.434 ± 3.538; *p* < 0.001) compared with non-TEE-guided individuals. Those in the TEE-guided group more often had lower left ventricular ejection fraction (LVEF) and higher New York Heart Assiocation (NYHA) functional class. Moreover, TEE-guided patients were characterized by lower blood levels of hemoglobin and were more frequently treated for chronic renal failure. The groups did not differ significantly in the incidence of other diseases such as diabetes mellitus, coronary artery disease, previous sternotomy or chronic atrial fibrillation, whereas arterial hypertension was more common in non-TEE-guided individuals (60.779 vs. 53.012; *p* < 0.001). Patient age at first CIED implantation was comparable between the groups: 58.096 ± 15.813 vs. 57.348 ± 17.628; *p* = 0.558, whereas patients in the TEE-guided group were older at the time of lead extraction (67.419 ± 14.232 vs. 64.849 ± 16.223; *p* < 0.001) (Table 1).

### 3.2. Preprocedural Data on CIED

Noninfectious indications for TLE were most frequent in the TEE-guided group (77.943% vs. 60.351%; *p* < 0.001), whereas infectious indications were more common in the non-TEE-guided group; both lead-related infective endocarditis (LRIE) and pocket infection were more frequent in patients without intraprocedural TEE (27.635% vs. 15.477%; *p* < 0.001 and 12.013% vs. 6.580%; *p* < 0.001, respectively). The number of implanted leads was similar in both groups. TEE-guided patients had older leads: the lead dwell time of the oldest leads before TLE was 112.665 ± 75.955 vs. 90.807 ± 69.959 months; *p* < 0.001. Furthermore, patients undergoing TLE under echocardiographic guidance had more ICDs or CSs (35.125% vs. 30.009%; *p* < 0.01), and fewer abandoned leads (0.087 ± 0.282 vs. 0.135 ± 0.342; *p* < 0.05) than those without intraprocedural TEE (Table 2).

### 3.3. Outcomes of Transvenous Lead Extraction

A total of 5258 leads were extracted, including 1760 in the TEE-guided group and 3498 in the non-TEE-guided group. In both groups, the number of extracted leads per patient was similar on average, 1.631 ± 0.719 vs. 1.661 ± 0.761; *p* = 0.477. The TEE-guided group differed from the non-TEE-guided group by the older age of the extracted leads (sum of lead dwell times: 15.466 ± 13.960 vs. 11.876 ± 11.004 years; *p* < 0.001), more frequent removal of ICD leads (31.603% vs. 25.309%; *p* < 0.001), less frequent extraction of left ventricular leads (11.307% vs. 14.008, *p* < 0.05) and abandoned leads (8.804% vs. 15.527%; *p* < 0.001). Technical difficulties during the procedures and additional tools for lead removal were more common in the TEE-guided group (23.818% vs. 15.907%; *p* < 0.001 and 6.951% vs. 5.128%; *p* < 0.05, respectively), whereas modification of venous access was more frequently necessary in non-TEE-guided individuals (5.558 vs. 1.668; *p* < 0.001). The duration of the procedure, measured by various parameters (skin-to-skin, sheath-to-sheath and mean duration of one lead extraction), was significantly longer in the TEE-guided group.

Complete procedural success was significantly higher in the TEE-guided group (97.683% vs. 95.442%, *p* < 0.01), whereas clinical success was comparable between the groups. In the periprocedural period, there was not a single death among TEE-guided patients, compared with six deaths (0.285%) in the non-TEE-guided group (*p* = 0.186) (Table 3).

### 3.4. Assessment of the Risk of Major Complications Using the SAFeTY TLE Score

The total score on the SAFeTY TLE scale was higher in the TEE-guided group (6.143 ± 4.395 vs. 5.593 ± 4.127; *p* = 0.004). The higher risk of major complications in this group was associated with longer lead dwell times and lower levels of hemoglobin (*p* < 0.001). According to the simulation, the risk of cardiac and venous perforation was significantly higher in individuals undergoing intraprocedural TEE monitoring: 1.898 (95% CI: 1.664–2.132) vs. 1.630 (95% CI: 1.472–1.788); *p* = 0.002. The SAFeTY TLE score yielded an expected number of 21 and 34 perforations in the superior vena cava (SVC), right atrium (RA) and right ventricle (RV) during TLE in the TEE-guided and non-TEE-guided group, respectively (Table 4).

**Figure 2:**
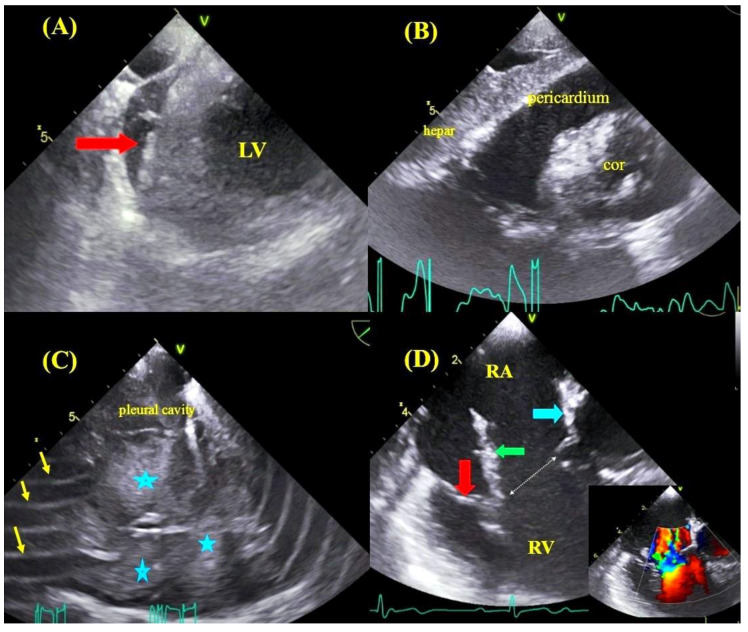
Possible TLE complications detected in TEE: TEE images—transgastric views, the formation of blood clot (red arrow) in the course of self-limiting bleeding into the pericardial sac (**A**), massive bleeding into the pericardial sac resulting in cardiac tamponade (**B**). TEE images—bleeding into the right pleural cavity with visible blood clots (asterisks), image blurring caused by electrocoagulation during thoracotomy (arrows) (**C**). TEE images—low esophageal view—injury to the tricuspid leaflets and papillary muscle rupture resulting in massive tricuspid regurgitation (color Doppler): anterior leaflet (red arrow), ruptured head of the papillary muscle (green arrow), fragment of the septal leaflet (blue arrow) (**D**).

The incidence of major complications did not differ significantly between the TEE-guided and non-TEE-guided group: 21 cases (1.946%) vs. 39 cases (1.852%), including 15 (1.390%) vs. 33 (1.567%) cases of hemorrhagic pericardial or pleural effusion and six (0.556%) vs. six (0.3285%) cases of significant damage to the tricuspid valve (Table 5, Figure 2).

Detailed analysis of major complications in both groups showed that, in TEE-guided patients, the number of major complications associated with hemorrhagic pericardial or pleural effusion was six less than predicted by the SAFeTY TLE score—a reduction of 28.57% (*p* < 0.05). In the non-TEE-guided group there were 33 major complications vs. 34 predicted by the risk calculator (Table 6).

## 4. Discussion

A search of the literature revealed few small observational studies and case reports that addressed the usefulness of transesophageal echocardiography for monitoring the patient during transvenous lead extraction [16,17,18,19,20]. This is the first study to compare the impact of TEE guidance on the effectiveness and safety of transvenous lead extraction procedures in two large groups: 1079 patients undergoing continuous TEE monitoring and 2106 patients with echocardiographic assessment before and after the procedure. The current study found that the use of continuous echocardiographic monitoring of lead extraction procedures resulted in a significantly decreased frequency of most dangerous complications related to cardiac and vascular damage.

**Figure 3:**
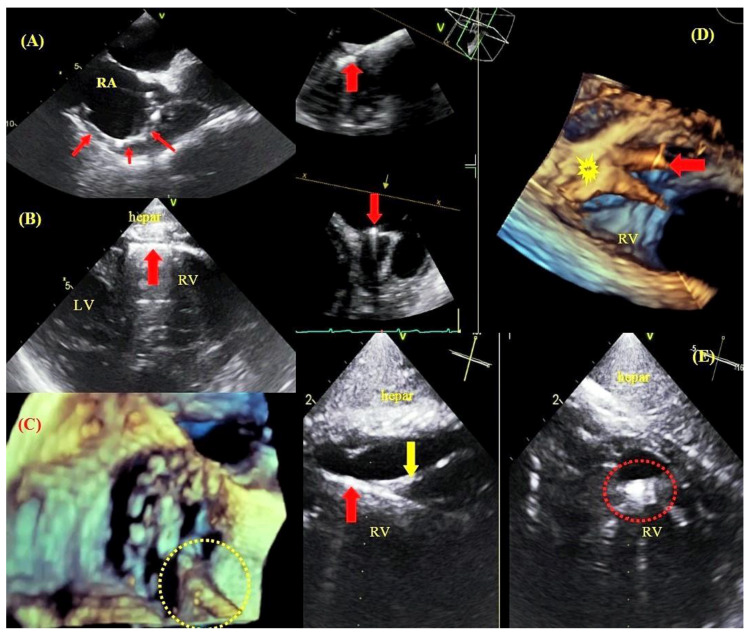
Binding sites between leads and cardiovascular structures visualized by TEE. TEE—bicaval view—a thickened lead (red arrows) adhered to the wall of the right atrium and the superior vena cava orifice (**A**). TEE—transgastric view—a ventricular lead (arrow) adhered to the right ventricular wall (**B**). 3D TEE imaging—the tricuspid valve with a lead adhered to the leaflet margin (**C**). 3D imaging (Multi-D)—a lead (red arrow) implanted at the base of the papillary muscle (yellow arrow) (**D**). TEE (Multi-D)—a ventricular lead (red arrow) adhered to the tendinous thread (yellow arrow) (**E**).

As is well known, age of the lead at the time of extraction is the most important risk factor for complications related to TLE. This parameter is incorporated into scoring systems for the prediction of technical difficulties during the procedure and for the stratification of the risk of major complications related to TLE [10,15]. Intraprocedural transesophageal echocardiography permits visualization and evaluation of structural changes caused by leads with long dwell times, thus facilitating navigation during the extraction procedure. In the initial phase of the procedure, it is very important to visualize lead adhesion to cardiac and vascular structures. TEE facilitates the imaging of lead adhesion to the walls of the superior vena cava, tricuspid valve and walls of the right atrium and right ventricle, as well as assessing lead-to-lead adhesion and the degree of fibrous tissue growth around lead tips in the cardiac wall (Figure 3).

The information provided by an echocardiographist during the procedure permits the extractor to take appropriate precautions during the freeing of the lead from the encapsulating fibrous tissue and pulling on the cardiac walls to remove the lead in its entirety.

**Figure 4:**
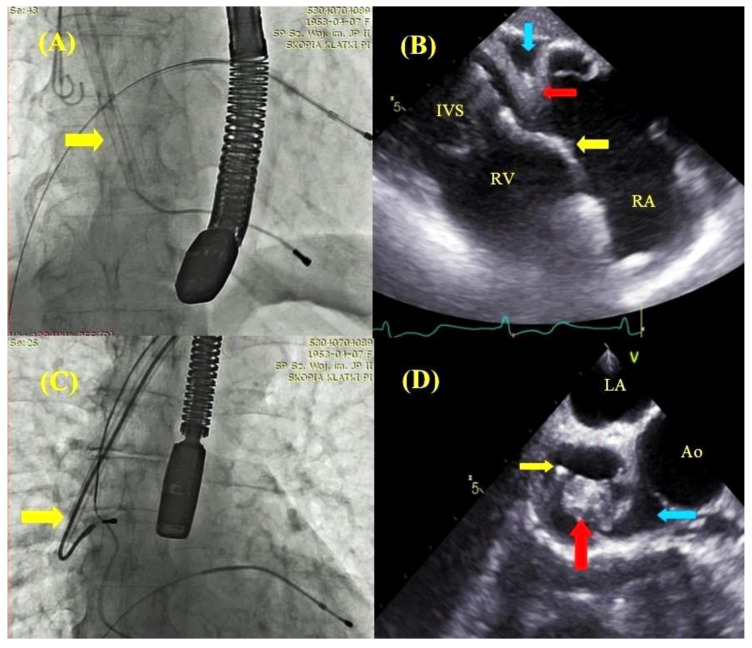
Comparison of fluoroscopic and echocardiographic images during lead extraction maneuvers. The moment of ventricular lead extracting; the yellow arrow points to the Byrd dilator slipped over the lead (**A**). TEE—transgastric view—the moment of ventricular lead extraction (**A**) with pulling on the right ventricular (RV) wall (red arrow), hyperechogenic thickened lead adhered to the endocardium (yellow arrow), the separation of pericardial layers corresponding to pseudo-cardiac tamponade (blue arrow) (**B**). The moment of atrial lead extraction; the yellow arrow points to the Byrd dilator slipped over the lead (**C**). TEE—mid-esophageal view; simultaneous atrial lead extraction (yellow arrow) (**C**), right atrial appendage prolapse into the atrial lumen (red arrow) and the separation of pericardial layers corresponding to pseudo-cardiac tamponade (blue arrow) (**D**).

**Figure 5:**
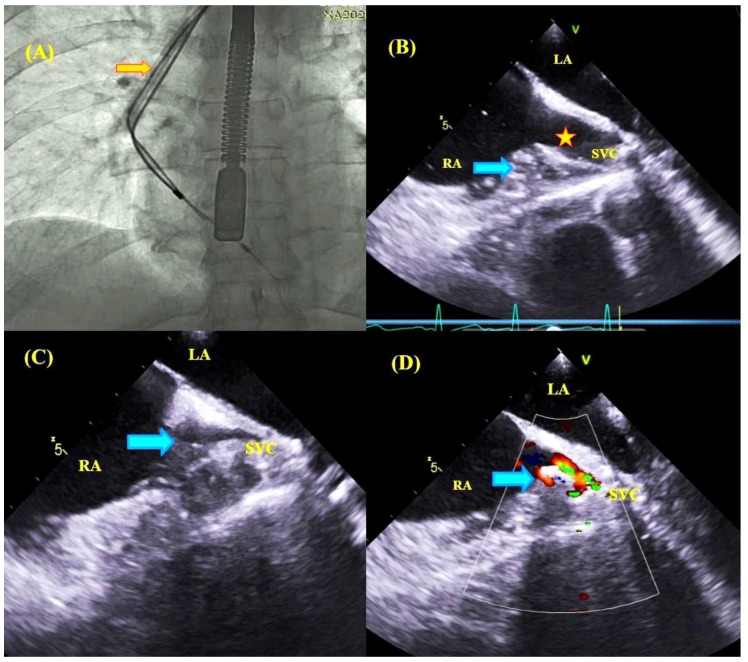
Comparison of fluoroscopic and TEE images during removal of lead-to-lead adhesions. Fluoroscopic image—the moment of ventricular lead extraction with simultaneous pulling on the atrial lead; the Byrd catheter slipped over the ventricular lead (orange arrow) (**A**). TEE images—mid-esophageal view, consecutive phases of pulling on the atrial wall and superior vena cava (blue arrows) until marked obliteration of the vessel during ventricular lead extraction and pulling on atrial lead adhesion (**B**–**D**).

The imaging of lead adhesions permitted us to determine the site of potential cardiac injury. An analysis of traction force and winding of soft tissues during dilator maneuvers warned the extractor of the risk of possible damage, which undoubtedly affected the procedure strategy, resulting in a reduction in the number of the most severe complications: cardiac and vascular perforations (Figure 4 and Figure 5).

A more detailed description of the echocardiographic findings in patients with TEE monitoring during TLE was presented in an earlier report [21].

The SAFeTY TLE score of 21 for wall perforations in the TEE-guided group was significantly higher than the number of actual complications (*n* = 15), which corresponds to a reduction of 28.571%; 95% CI (9.249–47.893); *p* = 0.028 in the risk of perforation. In the non-TEE-guided group, the score for perforations was 34 compared with the actual incidence of 33 complications, which confirms the accuracy of the calculation and significant benefits of using TEE as a monitoring tool during the extraction procedure. The decreased risk of damage to the superior vena cava, right atrium and right ventricle documented in patients undergoing TLE under continuous TEE guidance is certainly a result of implementing such an approach. This study supports evidence from previous small observational studies [16,17,18,19,20].

Apart from providing very important information on the challenges of lead extraction, continuous TEE monitoring allows for a prompt reaction to the occurrence of directly life-threatening complications: pericardial tamponade, hemorrhagic pleural effusion and tricuspid valve injury (Figure 2). It is interesting to note that a reduction in the most severe TLE complications was encountered in patients at potentially higher procedural risk. This study shows that patients with TEE guidance had older leads and were generally at higher risk of major complications according to the SAFeTY TLE score (6.143 ± 4.395 vs. 5.593 ± 4.127; *p* < 0.001) compared with non-TEE-guided procedures, which were characterized by more technical difficulties and prolonged operative time. Furthermore, patients with TEE guidance were older and presented with more comorbidities. Despite poorer clinical characteristics, complete procedural success was significantly higher in patients with TEE guidance (*p* < 0.01). The achievement of such a high rate of TLE effectiveness was also a result of echocardiographic navigation, because intraprocedural TEE frequently permitted continuation of the procedure in the situation of transient hemodynamic disorders (hypotension) associated with the traction of cardiac walls or superior vena cava (Figure 4 and Figure 5). In this way, it was possible to avoid tamponade, and at the same time to maintain the high level of operator alertness. In accordance with the present results, previous studies have demonstrated a similar impact of echocardiographic monitoring on TLE effectiveness, confirming that continuous echocardiographic monitoring allows for the completion of the extraction procedure in these circumstances [16,17,18].

**Figure 6:**
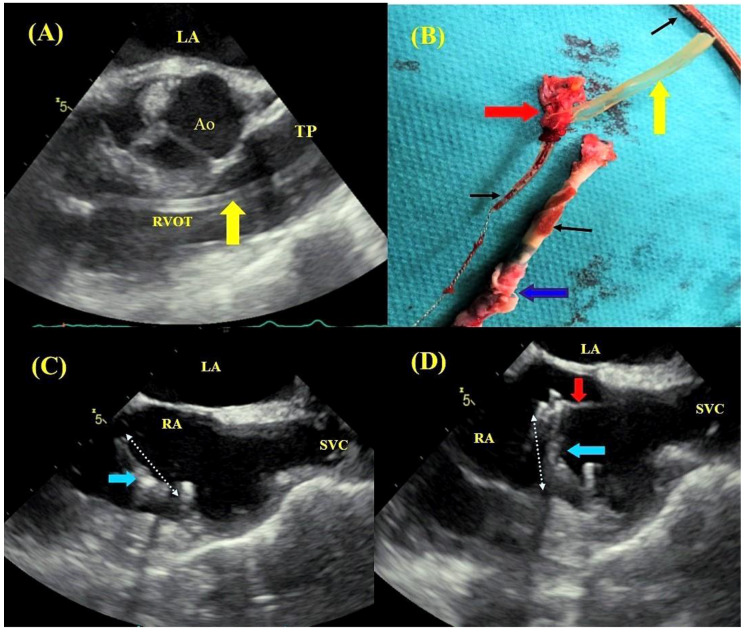
Remnants in heart chambers after TLE. TEE images—mid-esophageal view, transaortic view—fragment of silicone insulation (yellow arrow) stripped off the lead during TLE, dislodged into the right ventricular outflow tract (RVOT) and the pulmonary trunk (TP) (**A**). TEE images of the extracted ventricular lead (black arrows): unchanged fragment, lead tip with elongated guide wire, a fragment of endocardial tissue (red arrow) and silicone insulation (yellow arrow), segment of the lead surrounded by a fibrous capsule (blue arrow) and endocardial tissue (the image confirming lead adhesion to cardiac tissues) (**B**). TEE images—mid-esophageal bicaval view—fragment of a thickened (hyperechogenic) atrial lead adhering to the atrial wall, fractured during TLE (white and blue arrows) (**C**). The free-floating tip was captured by a lasso catheter (red arrow) (**D**).

Furthermore, it should be underscored that TEE imaging provides a direct assessment of lead fragments that may remain after the extraction. It is of paramount importance for procedure effectiveness to visualize lead fragments or insulation remnants, which are invisible in fluoroscopy (Figure 6).

This is especially important for the management of patients with infective endocarditis, in whom achievement of complete procedural success is associated with a better prognosis [13].

**Figure 7:**
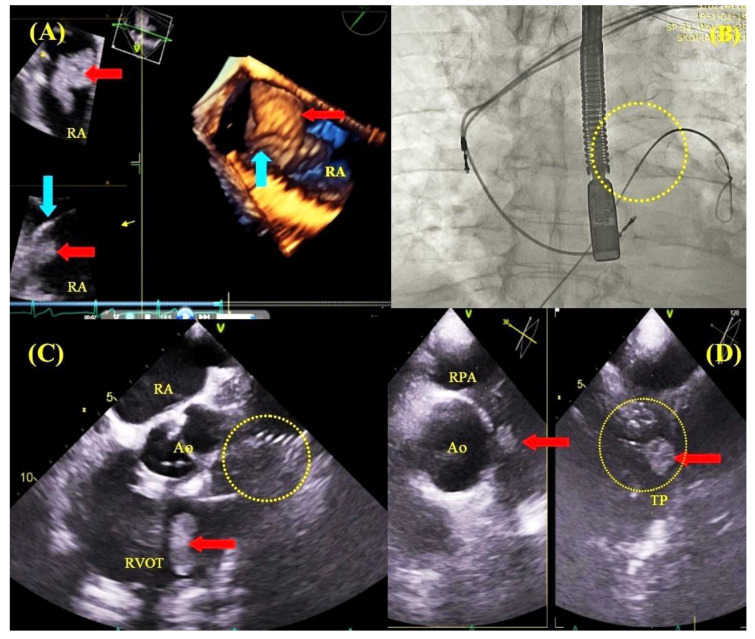
TLE in the course of lead-related infective endocarditis with pulmonary embolization protection devices and TEE guidance. 2D and D TEE images–a vegetation 4.0 × 1.7 cm in size (red arrow) on the ventricular lead (blue arrow) in the right atrium (**A**). Fluoroscopy–a nitinol basket (yellow circle) positioned within the pulmonary artery (pulmonary embolization protection), pulling on both leads (lead-to-lead adhesion) during atrial lead extraction (**B**). TEE images–mid-esophageal view, dislodgement of the vegetation (red arrow) into the RVOT after removal of the ventricular lead (**C**). TEE images–upper esophageal view (Multi D imaging), vegetation fragment captured by the basket in the pulmonary artery (**D**). However, it should be emphasized that there were no periprocedural deaths among TEE-guided patients, whereas six patients died among the non-TEE-guided individuals. In view of the above, this is the first study to document that use of continuous TEE monitoring in patients undergoing transvenous lead extraction, and it translates directly into a reduction in periprocedural mortality.

Similarly, it is important to visualize the migration of lead adhesions and vegetation, especially to the pulmonary artery. In these situations, TEE has an unquestionable advantage over fluoroscopy and is a valuable adjunct in the procedure of lead extraction (Figure 7) [10].

At short-term follow-up (6 and 12 months) survival after TLE was comparable between the groups. This is in accordance with earlier observations, which showed dissimilarity in risk factors for complications of TLE and factors influencing short- and long-term survival [5,11,12,22]. It is well known that survival after TLE is dependent mainly on clinical variables: patient age and comorbidities: diabetes mellitus, heart failure, renal failure. As previously stated, TEE-guided patients were characterized by older age and more comorbidities (Charlson comorbidity index 4.972 vs. 4.434; *p* < 0.001) compared with non-TEE-guided individuals, which must have contributed to the equalization of death rates between the two groups at follow-up.

## 5. Limitations

The organizational model of TLE procedures at the extraction centers was not the same and it did not take into account operator learning curves. Echocardiographic evaluation was not performed by one diagnostician. The study did not describe in detail the role of echocardiographic monitoring for the assessment of tricuspid valve function, as this parameter is not incorporated into the SAFeTY TLE scoring system.

## 6. Conclusions

This is the first study to confirm the higher procedural effectiveness and reduced incidence of cardiac tear and venous injury during transvenous lead extraction under transesophageal echocardiographic guidance. It was also shown that the use of continuous TEE monitoring was associated with a 100% periprocedural survival. Echocardiographic monitoring allows operators to perform many complex procedures safely and with more precision in patients at high procedural risk, through anticipating and thus preventing the occurrence of complications based on an analysis of changes in cardiac structures in response to the operator’s maneuvers, which are invisible in fluoroscopy. Such behavior also contributes to the modification of procedures and techniques in some cases, and consequently minimizes the risk of major complications.

## Figures and Tables

**Figure 1:**
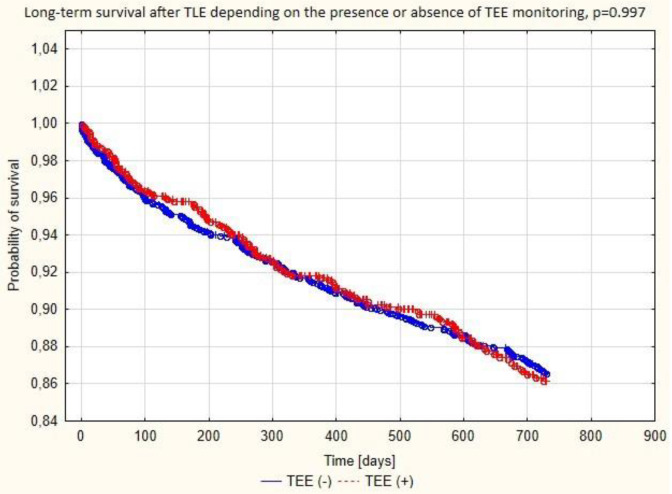
The Kaplan–Meier curve showing the probability of survival after transvenous lead extraction (TLE) in patients with and without transesophageal echocardiography (TEE) monitoring.

**Table 1:** Demographic and clinical information.

Parameter	All Group	With TEE Monitoring	Without TEE Monitoring	Mann–Whitney “U” Test/chi^2^ Test
Number of patients	3185	1079	2106	
Patient age during TLE (years); x ± SD	65.720 ± 15.622 N = 3185	67.419 ± 14.232 N = 1079	64.849 ± 16.223 N = 2106	*p* < 0.001
Patient age at first implantation (years); x ± SD	57.601 ± 17.036 N = 3185	58.096 ± 15.813 N = 1079	57.348 ± 17.628 N = 2106	*p* = 0.558
NYHA class; x ± SD	1.797 ± 0.692 N = 3185	2.035 ± 0.564 N = 1079	1.676 ± 0.719 N = 2106	*p* < 0.001
NYHA class III & IV; n (%)	451 (14.160) N = 3185	173 (16.033) N = 1079	278 (13.200) N = 2106	*p* < 0.05
LVEF [%]; x ± SD	48.911 ± 15.053 N = 3145	48.795 ± 15.852 N = 1070	48.971 ± 14.628 N = 2075	*p* = 0.896
LVEF < 40%; n (%)	879 (27.598) N = 3185	331 (30.677) N = 1079	548 (26.021) N = 2106	*p* < 0.01
Hemoglobin concentration; (g/dL 0 x ± SD	12.901 ± 1.946 N = 3121	12.589 ± 2.007 N = 1059	13.062 ± 1.894 N = 2062	*p* < 0.001
Renal failure, creatinine concentration > 1.3 mg/dL; n (%)	680 (21.601) N = 3148	262 (24.764) N = 1058	418 (20.000) N = 2090	*p* < 0.01
Diabetes mellitus; n (%)	624 (19.592) N = 3185	229 (21.223) N = 1079	395 (18.756) N = 2106	*p* = 0.107
Arterial hypertension; n (%)	1852 (58.148) N = 3185	572 (53.012) N = 1079	1280 (60.779) N = 2106	*p* < 0.001
Coronary artery disease/stroke/peripheral artery disease n; n (%)	1328 (41.695) N = 3185	455 (42.169) N = 1079	873 (41.453) N = 2106	*p* = 0.720
Permanent atrial fibrillation; n (%)	724 (22.732) N = 3185	257 (23.818) N = 1079	467 (22.175) N = 2106	*p* = 0.316
Prior sternotomy; n (%)	478 (15.008) N = 3185	145 (13.438) N = 1079	333 (15.812) N = 2106	*p* = 0.085
Charlson comorbidity index; x ± SD	4.616 ± 3.621 N = 3178	4.972 ± 3.756 N = 1072	4.434 ± 3.538 N = 2106	*p* < 0.001

Abbreviations: left ventricular ejection fraction (LVEF), N New York Heart Association (NYHSA), transvenous lead extraction (TLE).

**Table 2:** Indications for TLE and preoperative data on cardiac implantable electronic devices (CIED).

Parameter	All Group	With TEE Monitoring	Without TEE Monitoring	Mann–Whitney “U” Test/chi^2^ Test
Number of patients	3185	1079	2106	
**Indications for TLE**				
LRIE certain or probable with or without pocket infection; n (%)	749 (23.516) N = 3185	167 (15.477) N = 1079	582 (27.635) N = 2106	*p* <0.001
Local (pocket) infection (only); n (%)	324 (10.173) N = 3185	71 (6.580) N = 1079	253 (12.013) N = 2106	*p* < 0.001
Non-infectious indications, all; n (%)	2112 (66.311) N = 3185	841 (77.943) N = 1079	1271 (60.351) N = 2106	*p* < 0.001
* Mechanical lead damage (electric failure) n (%)	816 (38.636) N = 2112	337 (40.071) N = 841	479 (37.687) N = 1271	*p* = 0.291
* Lead dysfunction (exit/entry block, dislodgement, extracardiac pacing) n (%)	387 (18.324) N = 2112	159 (18.906) N = 841	228 (17.939) N = 1271	*p* = 0.614
* Lead dysfunction caused by (usually dry)—perforation n (%)	350 (16.572) N = 2112	143 (17.004) N = 841	207 (16.286) N = 1271	*p* = 0.708
* Change of pacing mode/upgrading, downgrading n (%)	168 (7.955) N = 2112	66 (7.848) N = 841	102 (8.025) N = 1271	*p* = 0.948
* Abandoned lead/prevention of abandonment (AF, overmuch of leads) n (%)	99 (4.688) N = 2112	28 (3.329) N = 841	71 (5.586) N = 1271	*p* < 0.05
* Threatened/potentially threatened lead (loops, free ending, left heart, LDTD) n (%)	89 (4.214)	36 (4.281) N = 841	53 (4.170) N = 1271	*p* = 0.989
* Other (MRI indication, cancer, pain of pocket, loss of indication for pacing / ICD) n (%)	72 (3.409) N = 2112	31 (3.686) N = 841	41 (3.226) N = 1271	*p* = 0.654
* Recapture venous access (symptomatic. occlusion, SVC syndrome, lead replacement/upgrading) n (%)	131 (6.203) N = 2112	41 (4.875) N = 841	90 (7.081) N = 1271	*p* < 0.05
**Preoperative data on CIED**				
Dwell time of oldest lead in patients before TLE (months); x ± SD	98.212 ± 72.773 N = 3185	112.665 ± 75.955 N = 1079	90.807 ± 69.959 N = 2106	*p* < 0.001
Mean implant duration before TLE (months); x ± SD	90.050 ± 63.656 N = 3185	105.235 ± 67.991 N = 1079	82.271 ± 59.858 N = 2106	*p* < 0.001
CIED with ICD or CS lead; n (%)	1011 (31.743) N = 3185	379 (35.125) N = 1079	632 (30.009) N = 2106	*p* < 0.01
Number of abandoned leads before TLE; x ± SD	0.119 ± 0.323 N = 3185	0.087 ± 0.282 N = 1079	0.135 ± 0.342 N = 2106	*p* < 0.05
Presence of abandoned lead(s) before TLE; n (%)	379 (11.900) N = 3185	95 (8.804) N = 1079	284 (13.485) N = 2106	*p* < 0.001
Abandoned leads only (EPM. EHV); n (%)	32 (1.004) N = 3185	4 (0.371) N = 1079	28 (1.330) N = 2106	*p* < 0.05
Number of leads in the system before TLE; x ± SD	1.817 ± 0.634 N = 3161	1.829 ± 0.622 N = 1076	1.812 ± 0.640 N = 2085	*p* = 0.400
Number of leads in the heart before TLE; x ± SD	1.962 ± 0.758 N = 3185	1.930 ± 0.715 N = 1079	1.978 ± 0.779 N = 2106	*p* = 0.324

* Main non-infectious indication. Abbreviations: atrial fibrillation (AF), cardiac implantable electronic device (CIED), coronary sinus (CS), defibrillator lead without previously removed unit (EHV), pacemaker lead without previously removed unit (EPM), implantable cardioverter-defibrillator (ICD), lead-related infective endocarditis (LRIE), lead-dependent tricuspid dysfunction (LDTD), magnetic resonance imaging (MRI).

**Table 3:** Data on TLE procedure.

Parameter	All Group	With TEE Monitoring	Without TEE Monitoring	Mann–Whitney “U” Test/chi^2^ Test
Number of patients	3185	1079	2106	
Number of extracted leads	5258	1760	3498	
Oldest extracted lead (years); x ± SD	8.021 ± 5.962 N = 3185	9.290 ± 6.307 N = 1079	7.372 ± 5.670 N = 2106	*p* < 0.001
Sum of dwell times of extracted leads (years); x ± SD	13.091 ± 12.202 N = 3185	15.466 ± 13.960 N = 1079	11.876 ± 11.004 N = 2106	*p* < 0.001
Number of extracted leads in one patient; x ± SD	1.651 ± 0.747 N = 3185	1.631 ± 0.719 N = 1079	1.661 ± 0.761 N = 2106	*p* = 0.477
HV therapy (ICD) lead was extracted; n (%)	874 (27.441) N = 3185	341 (31.603) N = 1079	533 (25.309) N = 2106	*p* < 0.001
CS (LV pacing) lead was extracted; n (%)	417 (13.093) N = 3185	122 (11.307) N = 1079	295 (14.008) N = 2106	*p* < 0.05
Extraction of abandoned lead(s) (any); n (%)	422 (13.250) N = 3185	95 (8.804) N = 1079	327 (15.527) N = 2106	*p* < 0.001
Lead fracture during extraction; n (%)	137 (4.301) N = 3185	51 (4.727) N = 1079	86 (4.084) N = 2106	*p* = 0.451
Loss of free lead fragment; n (%)	15 (0.471) N = 3185	7 (0.648) N = 1079	8 (0.380) N = 2106	*p* = 0.438
Technical difficulty during TLE (any); n (%)	592 (18.587) N = 3185	257 (23.818) N = 1079	335 (15.907) N = 2106	*p* < 0.001
Two or more technical difficulties; n (%)	127 (3.987) N = 3185	75 (6.951) N = 1079	52 (2.469) N = 2106	*p* < 0.001
Necessity to change venous approach; n (%)	135 (4.240) N = 3184	18 (1.668) N = 1079	117 (5.558) N = 2105	*p* < 0.001
Use of other than extracted lead venous approach; n (%)	173 (5.432) N = 3185	28 (2.595) N = 1079	145 (6.885) N = 2106	*p* < 0.001
Necessity to use other than Byrd dilator tools (Evo, TightR, lassos, basket catheters); n (%)	183 (5.746) N = 3185	75 (6.951) N = 1079	108 (5.128) N = 2106	*p* < 0.05
Procedure duration (skin-to-skin); minutes x ± SD	59.952 ± 25.717 N = 3185	62.747 ± 27.138 N = 1079	58.520 ± 24.842 N = 2106	*p* < 0.001
Procedure duration (sheath-to-sheath time); minutes x ± SD	15.125 ± 23.274 N = 3185	15.374 ± 24.396 N = 1079	14.997 ± 22.682 N = 2106	*p* < 0.001
Procedure duration average single lead extraction time; minutes x ± SD	8.965 ± 12.418 N = 3182	9.111 ± 14.260 N = 1079	8.891 ± 11.363 N = 2103	*p* < 0.01
Partial radiological success (retained tip or < 4 cm lead fragment); n (%)	136 (4.270) N = 3185	34 (3.151) N = 1079	102 (4.843) N = 2106	*p* < 0.05
Complete clinical success; n (%)	3116 (97.834) N = 3185	1056 (97.868) N = 1079	2060 (97.816) N = 2106	*p* = 0.975
Complete procedural success; n (%)	3064 (96.201) N = 3185	1054 (97.683) N = 1079	2010 (95.442) N = 2106	*p* < 0.01
Procedure-related death (intra-, post-procedural); n (%)	6 (0.188) N = 3185	0 (0.000) N = 1079	6 (0.285) N = 2106	*p* = 0.186
Died within 6 months; n (%)	166 5.212 N = 3185	48 (4.449) N = 1079	118 (5.603) N = 2106	*p* = 0.193
Died within one year; n (%)	263 (8.257) N = 3185	83 (7.692) N = 1079	180 (8.547) N = 2106	*p* = 0.446

Abbreviations: coronary sinus (CV), left ventricle (LV), high voltage (HV), transvenous lead extraction (TLE). Mortality at 6 and 12 months after TLE was similar in both groups (Figure 1).

**Table 4:** Simulation of major complications according to the SAFeTY TLE score in the validation cohort.

Simulation of Major Complications According to SAFeTY TLE Score		All Group	With TEE Monitoring	Without TEE Monitoring	Mann–Whitney “U” Test/chi^2^ Test
Number of patients		3185	1079	2106	
Sum of dwell times of extracted leads ≥ 16.5 years; n (%)	**S**	864 (27.127) N = 3185	360 (33.364) N = 1079	504 (23.932) (N = 2106	*p* < 0.001
Hemoglobin concentration ≤ 11.5 g/dL; n (%)	**A**	710 (22.749) N = 3121	299 (27.711) N = 1059	411 (19.932) N = 2062	*p* < 0.001
Female; n (%)	**Fe**	1231 (38.650) N = 3185	410 (37.998) N = 1079	821 (38.984) N = 2106	*p* = 0.616
Number of previous CIED procedures (all); x ± SD	**T**	1.822 ± 1.081 N = 3185	1.779 ± 0.965 N = 1079	1.844 ± 1.136 N = 2106	*p* = 0.852
Patients aged at first implantation below 30 years; n (%)	**Y**	275 (8.634) N = 3185	82 (7.600) N = 1079	193 (9.164) N = 2106	*p* = 0.155
Number of SAFeTY TLE score points; x ± SD		5.777 ± 4.226 N = 3165	6.143 ± 4.395 N = 1079	5.593 ± 4.127 N = 2106	0.004
Probability of perforation (SVC, RA, RV) according to SAFeTY-TLE score; x (95% CI)		1.721 95% CI (1.590–1.852)	1.898 95% CI (1.664–2.132)	1.630 95% CI (1.472–1.788)	0.002
Calculated number of major complications (perforations; SVC, RA, RV) according to SAFeTY-TLE score; n (%)		55 (1.721) N = 3185	21 (1.898) N = 1079	34 (1.898) N = 2106	0.592

Abbreviations: cardiac implantable electronic device (CIED), right atrium (RA), right ventricle (RV), superior vena cava (SVC).

**Table 5:** Number of real major complications.

Major Complication	All Group	With TEE Monitoring	Without TEE Monitoring	Mann–Whitney “U” Test/chi^2^ Test
Number of patients	3185	1079	2106	
Real major complication (any); n (%)	60 (1.884) N = 3185	21 (1.946) N = 1079	39 (1.852) N = 2106	0.962
Real hemopericardium; n (%)	43 (1.350) N = 3185	13 (1.205) N = 1079	30 (1.425) N = 2106	0.729
Real hemopericardium or hemothorax; n (%)	48 (1.507) N = 3185	15 (1.390) N = 1079	33 (1.567) N = 2106	0.815
Real tricuspid valve damage during TLE; n (%)	12 (0.377) N = 3185	6 (0.556) N = 1079	6 (0.285) N = 2106	0.381
Rescue cardiac surgery; n (%)	37 (1.162) N = 3185	16 (1.483) N = 1079	21 (0.997) N = 2106	*p* = 0.300

**Table 6:** Comparison of number of major complications (perforations; SVC, RA, RV), calculated vs real complications, according to data from Table 4 and Table 5.

	Number of Patients	Calculated Number of Major Complications (Perforations; SVC, RA, RV) According to SAFeTY-TLE Score; n (%)	Real Number of Major Complications (Perforations; SVC, RA, RV) n (%)	Change N (%)	chi^2^ Test
All group	N = 3185	55 (1.721) N = 3185	48 (1.507) N = 3185	−7 (−12.727)	*p* < 0.05
With TEE monitoring	N = 1079	21 (1.898) N = 1079	15 (1.390) N = 1079	−6 (−28.571)	*p* < 0.05
Without TEE monitoring	N = 2106	34 (1.898) N = 2106	33 (1.567) N = 2106	−1 (2.941)	*p* = 1.000

Abbreviations: superior vena cava (SVC), right atrium (RA) and right ventricle (RV); for the SAFeTY-TLE score, see Table 4.
